# Limited family members/staff communication in intensive care units in the Czech and Slovak Republics considerably increases anxiety in patients ´ relatives – the DEPRESS study

**DOI:** 10.1186/1471-244X-14-21

**Published:** 2014-01-27

**Authors:** Katerina Rusinova, Jaromir Kukal, Jiri Simek, Vladimir Cerny

**Affiliations:** 1 Department of Anesthesiology and Intensive Care Medicine, 1st Faculty of Medicine, Charles University in Prague and General University Hospital, Prague, Czech Republic; 2 Institute for Medical Humanities, First Faculty of Medicine, Charles University in Prague, Prague, Czech Republic; 3 Department of Software Engineering in Economy, Faculty of Nuclear Science and Physical Engineering, Czech Technical University, Prague, Czech Republic; 4 Department of Philosophy and Ethics in Helping Professions, Faculty of Health and Social studies, University of South Bohemia in Ceske Budejovice, Ceske Budejovice, Czech Republic; 5 Department of Anesthesia, Pain Management and Perioperative Medicine, Dalhousie University, Halifax, Canada; 6 Department of Anesthesiology and Intensive Care, Charles University in Prague, Faculty of Medicine in Hradec Kralove, University hospital in Hradec Kralove, Hradec Kralove, Czech Republic

## Abstract

**Background:**

Symptoms of anxiety and depression are common among family members of ICU patients and are culturally dependent. The aim of the study was to assess the prevalence of symptoms of anxiety and depression and associated factors in family members of ICU patients in two Central European countries.

**Methods:**

We conducted a prospective multicenter study involving 22 ICUs (250 beds) in the Czech and Slovak Republics. The Hospital Anxiety and Depression Scale (HADS) was used to assess symptoms of anxiety and depression in family members of ICU patients. Family member understanding of the patient’s condition was assessed using a structured interview and a questionnaire was used to assess satisfaction with family member/ICU staff communication.

**Results:**

Twenty two intensive care units (both adult and pediatric) in academic medical centers and community hospitals participated in the study. During a 6 month period, 405 family members of 293 patients were enrolled. We found a high prevalence of anxiety and depression symptoms – 78% and 54%, respectively. Information leaflets distributed to family members did not lower incidences of anxiety/depression. Family members with symptoms of depression reported higher levels of satisfaction according to the modified Critical Care Family Needs Inventory. Extended contact between staff and family members was the only related factor associated with anxiety reduction (p = 0.001).

**Conclusion:**

Family members of ICU patients in East European countries suffer from symptoms of anxiety and depression. We identified limited family member/ICU staff communication as an important health care professional-related factor associated with a higher incidence of symptoms of anxiety. This factor is potentially amenable to improvement and may serve as a target for proactive intervention proactive intervention.

## Background

Intensive care units are probably one of the most challenging and stressful places in a hospital in terms of anxiety and depression among family members of ICU patients.

Sizable amounts of quantitative and qualitative data regarding mental health symptoms are now available [1,2]. In large observational studies conducted in France, for example, Pochard et al. [3] found that 69% of family members had anxiety and 35% experienced depression early in their relative’s ICU stay, while 73% had anxiety and 35% had depression in the days preceding their relative’s ICU discharge or death [4]. In a cohort of cancer patients’ families, the prevalence of anxiety and depression was 71 and 50.3%, respectively [5]. These symptoms, as showed by Anderson et al. [6], diminish over time, but even at six months, 35% of families were still experiencing post-traumatic stress.

During the second half of the 20th century, for historical reasons, medical facilities and communication skills took different evolutionary paths in West and Central/East European countries. In Central and Eastern countries there was a steady increase in physician paternalism together with a decline in open communication between patients, families and medical staff [7]. Concomitantly, medical technologies progressed very slowly compared to Western countries.

The technological gap was closed rapidly in the early 1990s and after 1990, a patients’ rights codex was created and legislation concerning informed consent was modified and upgraded accordingly [8,9]. However, communication skills and interaction patterns between physicians and patients and/or relatives remained poorly analyzed in Central/East European countries and as such, any changes in these areas were difficult to evaluate.

As a consequence of the highly prevalent psychological distress (common in family members during a patients’ ICU stay) there has been growing interest and effort toward addressing this problem in the ICU (mainly studied in Western Europe & America [10-12] and poorly investigated in Central Europe). Realization of the importance of understanding the current patient/family member/ICU environment in Central/East European countries (where potential gaps may exist between advances in therapeutics in the ICU and meeting communication/psychosocial needs among family members) was the main rationale for this study, which was conducted in two Central European countries.

The aim was three-fold: first, to assess symptoms of anxiety and depression in family members of ICU patients; second, to determine how well family member understood the condition/situation of the involved patient (through a structured interview); and third, to specify family members’ needs and satisfaction (using a modified version of Molter’s Critical Care Family Needs Inventory) [13].

## Methods

### Study setting and study participants

We created the DEPRESS study working group for participating in data collection for the DEPRESS study (DEPRession and anxiEty in family memberS of ICU patientS in the Czech and Slovak Republics) which involved total of 22 participating ICUs in 13 university centers and 9 community hospitals (the list of participants is detailed in the Acknowledgment section).

The study recruited patients and family members of patients hospitalized in an ICU for more than 48 hours, between May and September 2007.

We defined ‘family members’ as all individuals who visited the patient in the ICU. All family members that visited a patient during the study period were potential participants. They were informed that a study focusing on their needs was ongoing and that they could participate in the study. Each family member could participate once during the study period. Family members were informed that returning the questionnaire to the attending physician and agreeing to answer the questions about their understanding of the involved patient’s condition, would constitute consent on their part.

The National Ethics Committee ruled that a returned questionnaire was sufficient to indicate consent (reference number 200703 S11P).

### Study procedures and measures

ICU characteristics: we documented the structure of each ICU, the existence of informational leaflets for family members, and whether the ICU had a written protocol for interacting with family members.

Patient characteristics: we registered each patients’ age, gender, marital status, occupation (for adult patients), the Knaus chronic health status score [14], and reasons for ICU admission. We included two scores assessing the gravity of their condition: the highest TISS (TISS = Therapeutic Intervention Scoring System [15] and the APACHE II score (Acute Physiology and Chronic Health Evaluation score), the latter being calculated within 24 hrs of ICU admission [16]. Length of ICU stay and clinical condition at discharge (living or deceased) on the last day of the study period were also recorded.

Family members were invited to fill in three questionnaires (i-iii) and to participate in a structured interview with a physician (iv).

i) The Hospital Anxiety and Depression Scale (HADS), a 14-item questionnaire (7 items for evaluation of anxiety, 7 for depression) with a cut-off scale of 10 [17] was used to assess symptoms of anxiety and depression.

ii) To evaluate the ability of ICUs to meet family needs, we used a modified version of Molter’s Critical Care Family Needs Inventory (CCFNI) [13].

iii) Family members were asked (using a questionnaire with yes/no questions) whether they had received contradictory information, were receiving support from their general practitioner, would like help from a psychologist, if information from the ICU staff was timely and appropriate, and whether they would like or would have liked to receive more information about the diagnosis, treatment, and prognosis of the involved patient.

iv) Comprehension of information provided by staff was checked using a structured interview performed by a physician who asked each family member about their comprehension of the reason(s) for admission, main treatment options, and prognosis of the patient (Additional file 1).

As with most similar ICU studies, we started the data collection 48 hours after patient admission and the completion of questionnaires, and the interview took place between the third and the last day of the patient’s ICU stay.

### Statistical analysis

Single real variables were described via sample, median and range (min-max). Data involving single binary variables were treated as belonging to a binomial distribution with unknown event probabilities; point estimates and confidence intervals were calculated. Event occurrences in two disjoined groups were investigated using an odds ratio related to logistic regression; point estimates and confidence intervals were calculated. A probability of p = 0.05 was considered statistically significant. Relationships between patients and family members were investigated as pairs (patient, family member). First, we performed an univariate analysis of our findings to assess the factors associated with anxiety and depression; followed by a stepwise multivariate forward-backward logistic regression to assess the effects of variables on anxiety and depression separately, as measured by the estimated odds ratio. Anxiety or depression (defined as a subscale score >10) was the dependent variable. Independent variables were the patient, family and ICU characteristics. Analysis was performed using MATLAB Statistics Toolbox (Mathworks Inc).

## Results

Twenty two ICUs (250 beds) participated in the study and interview report forms and questionnaires, completed by 405 family members, were analyzed. Twenty-one family members declined to participate in the study (reasons not documented).

### Characteristics of the 22 ICUs

Seventeen ICUs (78%) were adult and five (22%) were pediatric. All ICUs had a median of three senior physicians (range 1 – 10), a median of one resident (range 0 – 5), a nurse-to-patient ratio of 1.5 (range 0.75 – 2.5) and a median of 11.5 beds (range 5 – 21). The median time for daily visits was 3 hours (range 2 – 24). Ten ICUs (45.5%) had a specific or suitable room for ICU staff meetings with family members. In 11 ICUs (50%) family members received an information leaflet but only 1 ICU (4.5%) had a written protocol for interacting with family members.

The characteristics of patients and family members participating in the study are summarized in Table 1. The prevalence of anxiety and of depression in family members was 72.8% and 53.6%, respectively (Table 2). Factors associated with symptoms of anxiety and depression are presented in Table 3 (univariate logistic regression model) and Table 4 (multivariate logistic regression model).

**Table 1:** Characteristics of patients (n = 293) and family members (n = 405)

**Patient**	**Count (%) or median [range]**
Age	39 [0–87]
Female gender	112 (38.9)
Living alone	38 (19.7)
Unemployed	53 (26.4)
Primary admission to ICU	108 (37.2)
Secondary admission to ICU	180 (62.1)
Knaus score	1 [1–4]
Highest TISS score	49 [15–80]
APACHE II within first 24 hours	20 [2–42]
Length of stay [days]	13 [3–242]
Status at discharge (died)	28 (9.9)
- Adult patients (died)	26 (13.8)
- Pediatric patients (died)	2 (2.1)
**Family member**	
Age	41.5 [16–87]
Female gender	289 (71.5)
Relationship to the patient	
Spouse	92 (22.8)
Parents	194 (48)
Siblings	62 (15.3)
Other	30 (7.4)
Not relative FM	4 (1)
Time of transport to the hospital [min]	40 [10–440]
Desired number of visits	7 [2–8]
Wanted more information about diagnosis	212 (52)
Wanted more information about treatment	214 (53)
Wanted more information about prognosis	226 (56)
Receiving contradictory information	70 (17.4)
Length of staff contacts [min]	10 [1–60]
Wanted help from psychologist	110 (27.7)
Not helped by general practitioner	258 (64.8)
Ignoring specific role of health care professionals	51 (12.6)
Suitable duration of meeting with ICU staff [min]	10 [1–60]
CCFNI score	20 [14–36]
Failure to understand (diagnosis AND treatment AND prognosis)	248 (61.9)

**Table 2:** Prevalence of anxiety and/or depression in family members (N = 400)

**Count**	**Spouses**	**Family members except spouses**	**All family members**
**Prob. % [95%CI]**	**(n = 92)**	**(n = 308)**	**(n = 400)**
Anxiety	77	217	294
	83.7 [74–91]	70.5 [65–76]	73.5 [68–78]
Depression	64	153	217
	69.6 [59–79]	49.7 [43–56]	54.3 [49–60]
At least one (anxiety or depression)	81	234	315
	88.0 [78–94]	76 [70–81]	78.8 [74–83]
Both (anxiety and depression)	60	136	196
	65 [54–75]	44.2 [38–50]	49.0 [43–54]

CI, confidence interval.

**Table 3:** Factors associated with symptoms of anxiety and/or depression in family members in a an univariate logistic regression model

	**Odds ratio (95%CI)**	**Odds ratio (95%CI)**
	**For anxiety**	**For depression**
**Patient related**		
Living with/in a family	1.87 (1.15–3.04); p = 0.012	NS
Knaus score	0.78 (0.66–0.92) p = 0.003	NS
TISS score	1.010 (1.002–1.020) p = 0.017	1.008 (1.0004–1.016) p = 0.038
**Family related**		
Age	NS	1.021 (1.006–1.036) p = 0.005
Gender (male)	0.63 (0.39–0.99) p = 0.047	NS
Relation (NON spouse/parent/child)	0.68 (0.57–0.82) p = 0.0001	0.71 (0.60–0.85) p = 0.0002
Number of visits/week	1.28 (1.10–1.49) p = 0.001	NS
Desired number of visits	1.16 (1.05–1.29) p = 0.005	1.13 (1.02–1.24) p = 0.018
Wanted more information about disease	1.62 (1.04–2.52) p = 0.032	1.64 (1.11–2.44) p = 0.014
Wanted more information about treatment	NS	1.93 (1.30–2.87) p = 0.001
Wanted more information about prognosis	1.77 (1.14–2.76) p = 0.011	1.90 (1.28–2.84) p = 0.002
Wanted help from psychologist	2.66 (1.53–4.62) p = 0.0005	2.43 (1.55–3.81) p = 0.0001
**Health care professional related**		
Duration of the information provided	0.95 (0.92–0.98) p = 0.001	NS

**Table 4:** Factors associated with symptoms of anxiety and/or depression in family members in a multivariate logistic regression model

	**Odds ratio (95%CI)**	**Odds ratio (95%CI)**
	**For anxiety**	**For depression**
**Patient related**		
Age	NS	0.99 (0.98–0.998); *p = 0.01*
Living with/in a family	1.97 (1.15–3.27); *p* = 0.009	NS
Knauses	0.77 (0.65–0.91); *p = 0.002*	NS
TISS max	1.0147 (1.01–1.02); *p = 002*	1.02 (1.01–1.03); *p = 0.002*
**Family related**		
Age	NS	1.02 (1.01–1.04); *p = 0.002*
Relation (NON spouse, NON parent/child)	0.69 (0.57–0.83); *p = 001*	0.74 (0.62–0.89); *p = 0.001*
Time for transport to the hospital	0.996 (0.992–0.9994); *p = 002*	NS
Desire number of visits	123 (0.16–1.44); *p = 0.008*	NS
Wanted more information about prognosis	NS	1.86 (1.22–2.85); *p = 0.004*
Wanted help from psychologist	2.51 (1.41–445); *p = 0.002*	2.16 (1.35–3.45); *p = 0.001*
**Caregiver related**		
Duration of the information provided	0.95 (0.92–098); *p = 0.001*	NS

Anxiety was associated with three patient-related characteristics (living with/in the family, TISS max, Knaus score), four family-related factors (relation other than spouse/parent/child, driving time to the hospital, desired number of visits, desire for psychological support) and one health care professional-related factor (length of ICU staff/family member interactions related to patient information).

Factors associated with symptoms of depression included two patient-related characteristics (age, TISS score max) and three family-related characteristics (relation other than spouse/parent/child, desire for more information about the prognosis, desire for psychological support).

#### Patient related characteristics associated with symptoms of anxiety/depression

The age of the patient was inversely associated with a lower depression rate among family members. The fact that the patient was living with/in the family was associated with higher anxiety but did not influence depressive symptoms. Lower Knaus scores protected from anxiety manifestations and signs of severity of the patient’s condition measured using TISS scores clearly increased both anxiety and depression.

#### Family related characteristics associated with symptoms of anxiety/depression

The age of the family members was positively correlated with symptoms of depression. The degree of paternity affected anxiety and depression, i.e. more distant relationships (relation = NON spouse and NON parent/child) minimized the effect. Driving time to the hospital was inversely correlated with levels of anxiety, while the number of visits desired by family members was directly related to anxiety. Family members that wanted more information about the prognosis experienced more symptoms of depression. Desire for psychological support correlated with both anxiety and depression symptoms.

#### Health care professional-related characteristics associated with symptoms of anxiety/depression

Longer periods of communication with ICU staff were directly related to decreased anxiety.

A median of 20 points (range 14 – 36), on the modified version of Molter’s Critical Care Family Needs Inventory (CCFNI), was found for family members. Interestingly, family members with symptoms of depression reported higher levels of satisfaction (in terms of CCFNI questions, e.g. quality of communication with ICU staff, perceived quality of care, etc.) than those without depression (p = 0.002).

Seventy family members (17.4%) reported receiving contradictory information; 51 (12.6%) did not know the specific role of each health care professional; 110 (27.4%) wanted help from a psychologist; and 258 (64.8%) were not receiving assistance from their general practitioner. Family members reported that a median of 10 minutes (range 1 – 60) would be a suitable amount of time for family member/ICU staff communication.

A total of 248 family members (61.2%) didn’t understand the patient’s diagnosis, main features of treatment, and/or the prognosis. Significantly better overall comprehension was found among family members of pediatric patients (OR 1.688, CI (1.17 – 2.32), p = 0.008). No differences in anxiety or depression were found between family members of pediatric and adult ICU patients.

## Discussion and conclusions

The symptoms of anxiety among families of ICU patients are known to reach up to 75% in many countries in the world [3,5]. Our study differs from previously published data in other countries and the differences could reflect historical patterns in Central and East European countries in the second half of the 20th century: e.g. a preference for personal oral communication combined with a mistrust of written information, limited family member/ICU staff communication and depression being unexpectedly linked with higher reported satisfaction of family members.

Our first remarkable finding was that time dedicated to concise communication between health professionals and patients/relatives was perceived as being short. The FAMIREA investigators report 16 minutes as the median clocked time for providing information to families [18]. In our study, family members estimated the duration of physician communication to be less than 10 minutes. Furthermore, the length of information provided to family members was identified as the only health care professional-related factor linked to a lower incidence of symptoms of anxiety. Family members clearly preferred an extended educational style interview over receiving written information. This finding seems to mirror findings in a study focused on informed consent in the Czech Republic that reflected “an unquestioning willingness, of a significant proportion of citizens, to accept, in cases of illness, all decisions made by doctors during the course of treatment” [19].

**Figure 1:**
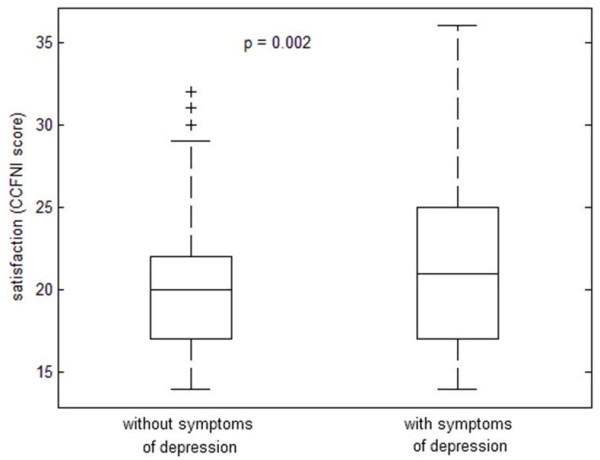
Family members’ satisfaction related to symptoms of depression.

Second, we found a higher prevalence of symptoms of depression, compared to the FAMIREA study (54% in our study vs. 35% in the French study [3]. Contrary to what might be expected, family members with symptoms of depression reported higher levels of satisfaction in terms of scoring on the modified CCFNI questionnaire (quality of medical care, understanding of provided information, staff member professionalism, patient visiting hours, quality of waiting rooms, and explanations regarding treatment and equipment) than those without depression (Figure 1). To our knowledge, this paradoxical finding does not have any parallel in the literature and is difficult to interpret. Why do family members report being more satisfied, while, at the same time, having higher scores related to symptoms of depression? We hypothesize that family members with symptoms of depression appreciate any information that helps them deal with emotionally traumatic situations. It probably does not provide appropriate assistance in coming to terms with grief or unfavorable information, but families may consider expressing any dissatisfaction as inappropriate in the context of the deep-rooted principle of free-of-charge medical care. Another hypothesis could be that their dissatisfaction was delayed, appearing only after discharge of the patient from the ICU.

Third, we observed that information leaflets distributed to family members were not linked to a lower incidence of anxiety/depression, which is in contrast to some previously published data [20,21]. The failure to be influenced by leaflets may suggest that many people still, perhaps subconsciously, distrust official written information.

All three above-mentioned points confirm that the relationship between patients and medical staff is changing much slower than medical technology. A genuine respect for patient autonomy combined with clear and honest communication with their relatives, recognized as cornerstones of a high-quality intensive care [22,23], are still at the periphery of medical staff attention in post-totalitarian countries; additionally, families are not offered sufficient opportunities to express emotions, voice concerns, and have questions answered.

Thus screening and appropriate referral becomes a critical step in mitigating these negative effects on the physical, mental and social functioning of family members.

Our study has several limitations. The collected data only reflect the circumstances in two Central European countries and thus should not be generalized. The selection of family members and patients was open to the discretion of the attending physician. This could potentially represent a selection bias in some centers. Additionally, we did not evaluate possible reasons for refusal of family members to participate in the study. And we also did not do family member follow-ups after discharge/death to assess the time course of the symptoms of anxiety and depression.

In conclusion, in our settings we identified limited family member/ICU staff communication as frequently associated with a higher incidence of symptoms of anxiety. This factor is potentially amenable to improvement and may serve as a target for proactive intervention.

## Abbreviations

(APACHE II): Acute Physiology and Chronic Health Evaluation score II score; (ICU): Intensive care unit; (FM): Family members; (HADS): Hospital Anxiety and Depression Scale; (CCFNI): Molter’s Critical Care Family Needs Inventory; (TISS): Therapeutic Intervention Scoring System.

## Competing interests

The authors declare that they have no competing interests.

## Authors' contributions

KR, JK and VC made substantial conceptual contributions to the design of the study, analysis of sources, and contributed to drafting of the manuscript. JK was involved in data analysis and statistical assessment. JS provided a critical revision of the manuscript regarding important intellectual content. KR and VC supplied important and relevant data from family members. All authors have read and approved the final version of the manuscript.

## Pre-publication history

The pre-publication history for this paper can be accessed here:

http://www.biomedcentral.com/1471-244X/14/21/prepub

## Supplementary Material

Additional file 1 Comprehension assessment. Click here for file

## References

[B1] Azoulay E Pochard F Kentish-Barnes N Chevret S Aboab J Adrie C Annane D Bleichner G Bollaert PE Darmon M Fassier T Galliot R Garrouste-Orgeas M Goulenok C Goldgran-Toledano D Hayon J Jourdain M Kaidomar M Laplace C Larché J Liotier J Papazian L Poisson C Reignier J Saidi F Schlemmer B FAMIREA Study Group Risk of post-traumatic stress symptoms in family members of intensive care unit patients Am J Respir Crit Care Med 2005 171 987 994 10.1164/rccm.200409-1295OC 15665319

[B2] Sprung CL Cohen SL Sjokvist P Baras M Bulow HH Hovilehto S Ledoux D Lippert A Maia P Phelan D Schobersberger W Wennberg E Woodcock T Ethicus Study Group End-of-life practices in European intensive care units: the Ethicus Study JAMA 2003 290 790 797 10.1001/jama.290.6.790 12915432

[B3] Pochard F Azoulay E Chevret S Lemaire F Hubert P Canoui P Grassin M Zittoun R Lle Gall JR Dhainaut JF Schlemmer B French FAMIREA Group Symptoms of anxiety and depression in family members of intensive care unit patients: Ethical hypothesis regarding decisionmaking capacity Crit Care Med 2001 29 1893 1897 10.1097/00003246-200110000-00007 11588447

[B4] Pochard F Darmon M Fassier T Symptoms of anxiety and depression in family members of intensive care unit patients before discharge or death. A prospective multicenter study J Crit Care 2005 20 90 96 10.1016/j.jcrc.2004.11.004 16015522

[B5] Fumis RR Deheinzelin D Family members of critically ill patients: assessing the symptoms of anxiety and depression Intensive Care Med 2009 35 899 902 10.1007/s00134-009-1406-7 19183953

[B6] Anderson WG Arnold RM Angus DC Bryce CL Posttraumatic stress and complicated grief in family members of patients in the intensive care unit J Gen Intern Med 2008 23 1871 1876 10.1007/s11606-008-0770-2 18780129 PMC2585673

[B7] Antonova P Jacobs DI Bojar M Cerný R Ciharová K Frick MA Fintel B DeHovitz J Bennett CL Czech health two decades on from the Velvet Revolution Lancet 2010 375 179 181 10.1016/S0140-6736(09)61293-9 19913289 PMC2925692

[B8] Haskovcova H Práva pacientu [The Rights of Patients] 1996 Krtilove, Havirov: Nakladatelstvi A

[B9] Simek J Krizová E Zamykalová J Informed Consent, Trust and Virtue in Czech Medicine Ethics, Law and Society, Volume IV 2009 London: Ashgate 237 246

[B10] Magnus VS Turkington L Communication interaction in ICU - Patient and staff experiences and perceptions Intensive Crit Care Nurs 2006 22 167 180 10.1016/j.iccn.2005.09.009 16298132

[B11] Curtis JR Engelberg RA Wenrich MD Nielsen EL Shannon SE Treece PD Tonelli MR Patrick DL Robins LS McGrath BB Rubenfeld GD Studying communication about end-of-life care during the ICU family conference: development of a framework J Crit Care 2002 17 147 160 10.1053/jcrc.2002.35929 12297990

[B12] Azoulay E Chevret S Leleu G One half of ICU ´s patient families experience inadequate communication with the physicians Crit Care Med 2000 28 3044 3049 10.1097/00003246-200008000-00061 10966293

[B13] Johnson D Wilson M Cavanaugh B Bryden C Gudmundson D Moodley O Measuring the ability to meet family needs in an intensive care unit Crit Care Med 1998 26 266 271 10.1097/00003246-199802000-00023 9468163

[B14] Knaus WA Draper EA Wagner DP Zimmerman JE Apache II: a severity of disease classification system Crit Care Med 1981 13 818 829 3928249

[B15] Cullen DJ Civetta JM Briggs BA Ferrara LC Therapeutic intervention scoring system: a method for quantitative comparison of patient care Crit Care Med 1974 2 57 60 10.1097/00003246-197403000-00001 4832281

[B16] Knaus WA Draper EA Wagner DP Zimmerman JE APACHE II: a severity of disease classification system Crit Care Med 1985 13 818 829 10.1097/00003246-198510000-00009 3928249

[B17] Bjelland I Dahl AA Haug TT Neckelmann D The validity of the Hospital Anxiety and Depression Scale. An updated literature review J Psychosom Res 2002 52 69 77 10.1016/S0022-3999(01)00296-3 11832252

[B18] Fassier T Darmon M Laplace C Chevret S Schlemmer B Pochard F Azoulay E One-day quantitative cross-sectional study of family information time in 90 intensive care units in France Crit Care Med 2007 35 177 183 10.1097/01.CCM.0000249834.26847.BE 17079999

[B19] Křížová E Šimek J Theory and practice of informed consent in the Czech Republic J Med Ethics 2007 33 273 277 10.1136/jme.2005.015164 17470503 PMC2598120

[B20] Lautrette A Darmon M Megarbane B Joly LM Chevret S Adrie C Barnoud D Bleichner G Bruel C Choukroun G Curtis JR Fieux F Galliot R Garrouste Orgeas M Georges H Goldgran Toledano D Jourdain M Loubert G Reignier J Saidi F Souweine B Vincent F Barnes NK Pochard F Schlemmer B Azoulay E A communication strategy and brochure for relatives of patients dying in the ICU N Engl J Med 2007 356 469 478 10.1056/NEJMoa063446 17267907

[B21] Azoulay E Pochard F Chevret S Jourdain M Bornstain C Wernet A Cattaneo I Annane D Brun F Bollaert PE Zahar JR Goldgran-Toledano D Adrie C Joly LM Tayoro J Desmettre T Pigne E Parrot A Sanchez O Poisson C Le Gall JR Schlemmer B Lemaire F Impact of a family information leaflet on effectiveness of information provided to family members of intensive care unit patients: a multicenter, prospective, randomized, controlled trial Am J Respir Crit Care Med 2002 165 438 442 10.1164/ajrccm.165.4.200108-006oc 11850333

[B22] Truog RD Campbell ML Curtis JR Haas CE Luce JM Rubenfeld GD Rushton CH Kaufman DC American Academy of Critical Care Medicine Recommendations for end-of-life care in the intensive care unit: a consensus statement by the American College [corrected] of Critical Care Medicine Crit Care Med 2008 36 953 963 10.1097/CCM.0B013E3181659096 18431285

[B23] Curtis JR Patrick DL Shannon SE Treece PD Engelberg RA Rubenfeld GD The family conference as a focus to improve communication about end-of-life care in the intensive care unit: opportunities for improvement Crit Care Med 2001 29 Suppl N26 N33 11228570 10.1097/00003246-200102001-00006

