# Supplementary material for: Limited family members/staff communication in intensive care units in the Czech and Slovak Republics considerably increases anxiety in patients ´ relatives – the DEPRESS study
Source: BMC Psychiatry. 2014 Jan 27;14:21. doi: 10.1186/1471-244X-14-21 (PMC3931312; doi:10.1186/1471-244X-14-21)
Supplement: Additional file 1 — Comprehension assessment. [file 1471-244X-14-21-S1.docx]

**Additional file: comprehension assessment**

**Center: [_][_]** DEPRESS STUDY: Comprehension assessment

**PACIENT: [_][_]**

**Family member No. [_]**

**Day of ICU stay [_][_]**

1. **Diagnosis comprehension:**

*„Have you understand the reason for admission of your relative to the intensive care unit ?*

Evaluation: YES, if the family member correctly names the main organ involved in the acute illness or describes the diagnosis in his/herown words. Otherwise, code NO.

ANSWER: (YES = 1, NO = 0) ⎢_⎢

1. Prognosis comprehension

*« Have you understand the gravity of your relative’s condition ? What is his chance to recover (or survive) ?»*

Evaluation: YES, if the family member is able clearly to express if the prognosis is rather positive or rather negative.

ANSWER: (YES = 1, NO = 0) ⎢_⎢

1. Treatment comprehension

*« Do you know the main treatment methods and procedures in your relative ? »*

The investigator first checks the ongoing treatment and than fills in the family members answer.

| **Treatment** | **Physician** | **Familymember** |
| --- | --- | --- |
|  |  |  |
| Invasive/noninvasive ventilation | ⎢_⎢ | ⎢_⎢ |
| oxygen | ⎢_⎢ | ⎢_⎢ |
| Nebulisation | ⎢_⎢ | ⎢_⎢ |
| Inotropic drugs | ⎢_⎢ | ⎢_⎢ |
| Analgesia/sedation | ⎢_⎢ | ⎢_⎢ |
| Renal replacement therapy | ⎢_⎢ | ⎢_⎢ |
| Thoracic drainage | ⎢_⎢ | ⎢_⎢ |
| Artificial nutrition (enteral/parenteral) | ⎢_⎢ | ⎢_⎢ |
| Transfusions | ⎢_⎢ | ⎢_⎢ |
| Operations, cannulations, wounds | ⎢_⎢ | ⎢_⎢ |
| Antibiotics | ⎢_⎢ | ⎢_⎢ |
